# Herb-Induced Liver Injury: Phylogenetic Relationship, Structure-Toxicity Relationship, and Herb-Ingredient Network Analysis

**DOI:** 10.3390/ijms20153633

**Published:** 2019-07-25

**Authors:** Shuaibing He, Chenyang Zhang, Ping Zhou, Xuelian Zhang, Tianyuan Ye, Ruiying Wang, Guibo Sun, Xiaobo Sun

**Affiliations:** 1 Beijing Key Laboratory of Innovative Drug Discovery of Traditional Chinese Medicine (Natural Medicine) and Translational Medicine, Institute of Medicinal Plant Development, Peking Union Medical College and Chinese Academy of Medical Sciences, Beijing 100193, China; 2 Key Laboratory of Bioactive Substances and Resource Utilization of Chinese Herbal Medicine, Ministry of Education, Beijing 100193, China; 3 Key Laboratory of Efficacy Evaluation of Chinese Medicine against Glycolipid Metabolic Disorders, State Administration of Traditional Chinese Medicine, Beijing 100193, China; 4 Key Laboratory of new drug discovery based on Classic Chinese medicine prescription, Chinese Academy of Medical Sciences, Beijing 100193, China

**Keywords:** herb, hepatotoxicity, HILI, phylogenetic tree, structure-toxicity relationship, herb-ingredient network, mechanism

## Abstract

Currently, hundreds of herbal products with potential hepatotoxicity were available in the literature. A comprehensive summary and analysis focused on these potential hepatotoxic herbal products may assist in understanding herb-induced liver injury (HILI). In this work, we collected 335 hepatotoxic medicinal plants, 296 hepatotoxic ingredients, and 584 hepatoprotective ingredients through a systematic literature retrieval. Then we analyzed these data from the perspectives of phylogenetic relationship and structure-toxicity relationship. Phylogenetic analysis indicated that hepatotoxic medicinal plants tended to have a closer taxonomic relationship. By investigating the structures of the hepatotoxic ingredients, we found that alkaloids and terpenoids were the two major groups of hepatotoxicity. We also identified eight major skeletons of hepatotoxicity and reviewed their hepatotoxic mechanisms. Additionally, 15 structural alerts (SAs) for hepatotoxicity were identified based on SARpy software. These SAs will help to estimate the hepatotoxic risk of ingredients from herbs. Finally, a herb-ingredient network was constructed by integrating multiple datasets, which will assist to identify the hepatotoxic ingredients of herb/herb-formula quickly. In summary, a systemic analysis focused on HILI was conducted which will not only assist to identify the toxic molecular basis of hepatotoxic herbs but also contribute to decipher the mechanisms of HILI.

## 1. Introduction

As the major organ of drug metabolism, liver is more likely to suffer from drug injury than other organs. Drug-induced liver injury (DILI) always leads to severe clinical adverse events, including hepatitis, liver fibrosis, liver failure, and even death [1,2,3]. Currently, more than 1100 medicines have been reported to cause various degrees of liver toxicity [4]. Data from the United States indicated that DILI has become the leading cause of acute liver injury events in clinical [5]. In addition, among 14 toxicity factors of drug withdrawal from markets, DILI ranking first by a percentage of 27% [6]. Therefore, it is of significance to study DILI.

The use of herbs in treating diseases has a history of thousands of years in many Asian countries. During the past decades, herbs or their extracts have gained increasing attention worldwide due to their significant efficacy in treating and preventing some diseases [7,8,9,10,11]. The most typical example is artemisinin, which was first discovered by the Chinese scientist Prof. You-you Tu and her team from the plant *Artemisia annua* (a herb which was commonly prescribed for hemorrhoids and malaria in Chinese). For its significant efficacy in the prevention and treatment of malaria, artemisinin was widely recognized by the world. Due to this important discovery, Prof. You-you Tu was awarded the 2015 Nobel Prize in Physiology or Medicine [12]. In recent years, artemisinin was found to also treat schizophrenic disorders for which the Phase III clinical trial has been completed [13]. Currently, it was reported that 4 billion people, about 80% of the world’s population consumed herbs to treat various diseases [14]. In contrast to synthetic drugs, herbs were commonly thought to be safe and harmless, as they come from nature. It was roughly estimated that more than 500 herbal products were consumed as harmless health products worldwide [15]. However, like synthetic drugs, herbs also possess adverse effects. In recent years, more and more clinical cases and laboratory data have demonstrated that some herbal products may cause varying degrees of liver damage. In a recent report, the etiology of DILI in Mainland China was investigated based on 25927 DILI cases from 308 medical centers. As a result, herbal and dietary supplements ranking first among 11 single classes of implicated drugs (26.81%) of DILI [16]. In another research from Korea in 2019, twenty-five percent of the DILI cases were attributed to herbals [17]. Herb-induced liver injury (HILI) is not restricted to China, Japan, and Korea but commonly observed in all countries that consume herbs. Cases mentioned above showed that HILI is a serious public health problem and deserves more attention. 

Several factors as follows were documented to be associated with HILI. (1) The gender, age, organism state, and genetic background of patients [18,19,20,21], (2) the dose and course of treatment of herbs [22,23], (3) the metabolism, misuse, and abuse of herbs [24,25], and (4) the quality of herbs, including adulterated products, bacterial contamination, the presence of heavy metals, pesticides, or solvents [26,27,28,29]. Additionally, drug interaction may be another risk factor of HILI [30,31]. Depending on the type of target cells suffered from damage, DILI can be generally divided into three types: cholestatic liver injury, hepatocellular liver injury, and mixed liver injury (hepatocellular and cholestatic) [32]. According to the detail pathological features, some academics even categorized DILI into 21 types [33]. In the light of the pathogenesis of liver injury, hepatotoxic agents can be classified into two groups: direct and indirect hepatotoxic agents. Direct hepatotoxic agents are substances that cause cholestasis by intervening the bile secretion process. In contrast to direct hepatotoxic agents, indirect hepatotoxic agents induced liver injury via producing selective metabolic blocks or physiologic lesions. These lesions always lead to the disruption of some essential metabolic pathways and cellular biochemical processes [34,35]. Numerous influential risk factors and extensive disease spectrum increased the complexity and diversity of the mechanisms of DILI. Currently, hepatotoxic mechanisms for most herbs are far from elucidation. Herb is not a single chemical entity but a complex mixture of both beneficial and toxic ingredients. Therefore, identifying toxic ingredients may be the first step to understanding the exact mechanisms of HILI. During the past decades, hundreds of herbs/herbal extracts were reported to cause varying degrees of liver damage. However, until now, a systemic review focused on these hepatotoxic substances was still unavailable, which prevented academics and pharmacologists from understanding the occurrence of HILI comprehensively. Therefore, in this work, we summarized and analyzed the hepatotoxic herbs/ingredients published in the literature by which we attempted to find some clues about the occurrence of HILI from the perspectives of the phylogenetic relationship and structure-toxicity relationship. 

## 2. Results and Discussion

### 2.1. Cluster Pattern of Medicinal Plants with Potential Hepatotoxicity

In this work, we collected 335 medicinal plants with potential hepatotoxicity. A total of 219 genera, 103 families, and 46 orders were involved (Supplementary file 1). The majority of these species (*n* = 323, 96.42%) belonged to the mesangiospermae clade. In order scale, a total of 12 orders containing hepatotoxicity species > 10. These 12 orders accounted for 68.66% (*n* = 230) of the hepatotoxic species (Table 1). In terms of the probability of hepatotoxicity, the Top-5 orders were Ranunculales, Boraginales, Piperales, Sapindales, and Apiales, respectively. Medicinal plants within these five orders may be faced with higher hepatotoxic risk. Specially, as the leading productive order of hepatotoxic herbs, Ranunculales should be given more attention in clinical application. 

In family scale, a total of 104 families were involved. About half (*n* = 48, 46.60%) of these families only included 1 hepatotoxic member. Table 2 showed the Top-10 most productive families of hepatotoxic herbs. They accounted for 42.69% (*n* = 143) of the hepatotoxic herbs. Among these 10 families, Aristolochiaceae is most likely to suffer from hepatotoxicity followed by Polygonaceae, Ranunculaceae, Boraginaceae, and Lamiaceae, respectively.

In genus scale, a total of 219 genera were involved. However, only 68 (31.05%) genera contained more than 2 hepatotoxic members. As listed in Table 3, a total of 15 genera containing hepatotoxic herbs greater than 3.

In summary, hepatotoxic botanicals were widely distributed in nature. As demonstrated in Figures S1–S3 (Supplementary file 2), the hepatotoxic botanicals were not scattered equally in phylogenetic tree, but displayed significant cluster pattern. Hepatotoxic medicinal plants tended to have a closer taxonomic relationship. Therefore, we can claim that there is a certain degree of correlation between the hepatotoxicity of medicinal plants and their phylogenetic relationship. In other words, medicinal plants close to hepatotoxic botanicals may be confronted with higher hepatotoxic risk.

### 2.2. Phytochemicals with Potential Hepatotoxicity

#### 2.2.1. Skeleton-Based Cluster Pattern of Hepatotoxic Ingredients

**Figure 1:**
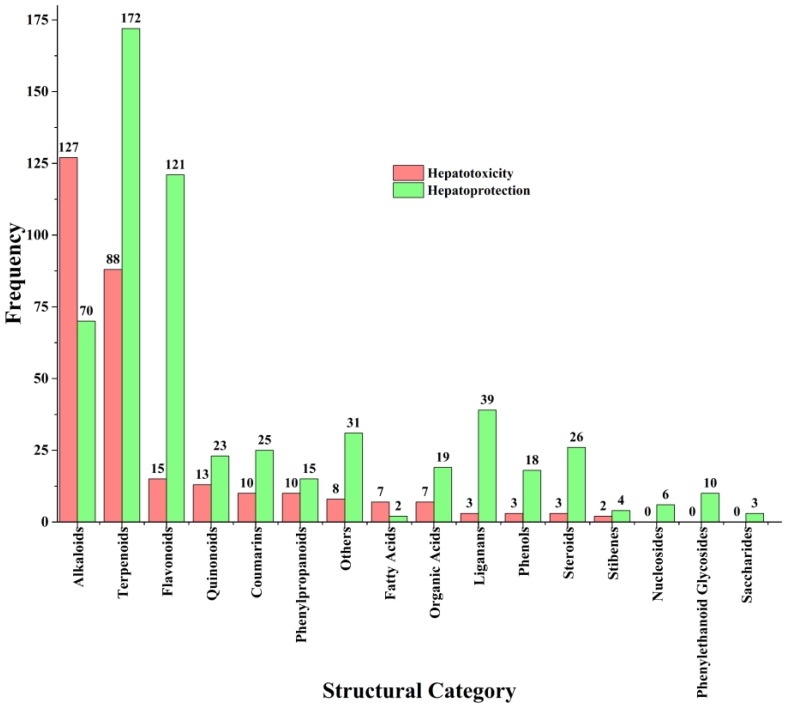
The structural distribution of ingredients with potential hepatotoxicity or hepatoprotection.

A total of 296 hepatotoxic ingredients and 584 hepatoprotective ingredients were collected (Supplementary file 3). Depending on the skeleton of each ingredient, we divided these ingredients into 17 categories (Figure 1). As the dominant categories of hepatotoxic ingredients, the frequencies of alkaloids and terpenoids were 127 (42.91%) and 88 (29.73%), respectively. The third, fourth, fifth, and sixth were flavonoids (14), quinones (13), coumarins (10), and phenylpropanoids (10), respectively. As the leading sub-category of alkaloids, pyrrolizidines accounted for 60.63% (*n* = 77) of the hepatotoxic alkaloids. Another two major sub-categories of alkaloids were isoquinolines (12) and benzophenanthridines (4), respectively. For terpenoids, the frequencies of the sub-categories decreased from sesquiterpenes (31) to triterpenes (17). The frequencies of monoterpenes and diterpenes were 20 and 19, respectively. Monoterpenes displayed significant structure-toxicity relationship. Thirteen of them belonged to the p-menthane type monoterpenes. Guaianes (9), cadinanes (4), eudesmanes (7), and germacranes (3) were the major sub-categories of sesquiterpenes. For triterpenes, three major sub-categories were oleananes (7), eupnanes (3), and meliacanes (3), respectively.

Several subcategories of flavonoids were involved, including flavones (4), flavanones (3), isoflavanones (2), isoflavones (1), flavonols (1), flavanonols (1), chalcones (1), flavanols (1), and xanthones (1). As natural polyphenols, flavonoids were abundant in natural products and widely consumed worldwide. Many flavonoids have shown significant efficacy in alleviating and preventing various chronic diseases [36]. Currently, more than 121 flavonoids have been reported to possess potential hepatoprotection effect. However, only 15 flavonoids were reported to cause liver damage. Therefore, we guessed that flavonoids may be faced with lower hepatotoxic risk. The frequency of emodin-type anthraquinone derivatives (*n* = 9, hydroxyl groups are attached to either sides of the anthraquinone nucleus) was significantly higher than that of alizarin-type anthraquinones (*n* = 2, hydroxyl groups are only attached to one side of the anthraquinone nucleus), indicating that the location of hydroxyl groups may be an important factor to assess the hepatotoxic risk of anthraquinones. Compared to simple coumarins (3/12), furanocoumarins displayed a higher ratio of hepatotoxic to non-hepatotoxic (6/5). According to the type of the C-3 side chain, simple phenylpropanooids generally can be categorized into four groups: phenylpropenes, phenylpropanols, phenylpropionaldehyds, and phenylpropionic acids. Herein, a total of 10 simple phenylpropanooids were reported to show potential hepatotoxicity among which nine belonged to the phenylpropenes. Therefore, we can claim that phenylpropenes may be a high risk group of hepatotoxicity.

As important natural products, alkaloids and terpenoids played essential roles in the development of novel drugs. Figure 1 showed that alkaloids (70) and terpenoids (172) may be the major and important natural sources of liver protectants. However, in this work, we also found that these two categories of phytochemicals were the high-incidence groups of hepatotoxicity. Therefore, hepatotoxic risk assessment focused on these two groups in the early stage of drug discovery should be given more attention. Additionally, flavonoids (121), liganans (39), steroids (26), and phenols (18) were found to contain abundant hepatoprotective ingredients. Importantly, liver adverse effects for these four groups were rarely reported. The number of hepatotoxic ingredients collected in this work was significantly less than that of hepatoprotective ingredients. Publication bias may be one of the reasons for such a situation. In general, some researchers were more inclined to report the beneficial effects of drug candidates than their toxic effects. In addition, the selection of research direction of academics may be another factor. After all, the initial objective of pharmaceutical research is not to detect the toxic effects of natural products but to discover their beneficial effects. In Section 2.1, a total of 335 hepatotoxic medicinal plants were collected. Apparently, the number of hepatotoxic ingredients was significantly less than that of hepatotoxic botanicals. Although there may exist some hepatotoxic ingredients escaped from our vision, it was still indicated that the toxic molecular basis of hepatotoxic herbs is far from elucidation.

#### 2.2.2. Chemical Space and Drug-Likeness

**Figure 2:**
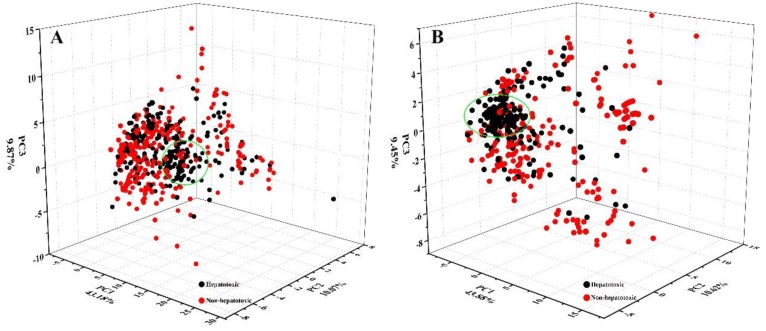
The chemical spaces of hepatotoxic and non-hepatotoxic ingredients.

Quantitative structure-activity relationship (QSAR) is an effective method to discover the relationship between structure and activity. During the past decades, QSAR has been successfully applied to screen drug candidates with various activities [37,38,39]. To investigate that whether it is feasible to measure the hepatotoxic risk of natural products by the QSAR methods, we investigated the distribution of hepatotoxic and non-hepatotoxic ingredients in the chemical space. Based on 84 physicochemical properties provided by Marvin (https://chemaxon.com/), we mapped hepatotoxic and non-hepatotoxic ingredients into three-dimensional chemical space by principal component analysis (PCA) method. Given that the number of non-hepatotoxic ingredients was significantly greater than that of hepatotoxic ingredients, Kennard-Stone algorithm [40] (a commonly used sample partition method) was adopted to balance the hepatotoxic dataset and non-hepatotoxic dataset. As a result, a total of 592 ingredients (Hepatotoxic: non-hepatotoxic = 1:1) were mapped into the chemical space. In Figure 2A, although a significant cluster (green circle) for hepatotoxicity was identified, it was really difficult to develop a hyperplane to differentiate hepatotoxic and non-hepatotoxic ingredients. Actually, 79 out of the hepatotoxic ingredients displayed biphasic effects to the liver, including liver protection and liver injury. These 79 ingredients may bring noise into the chemical space and increase the difficulty to differentiate hepatotoxic and non-hepatotoxic ingredients. After removing these 79 ingredients from the hepatotoxic dataset, we reinvestigated the distribution of hepatotoxic and non-hepatotoxic ingredients in the chemical space by the same method adopted above (Figure 2B). Consequently, most of the hepatotoxic ingredients were clustered into the green ellipse region. Inversely, non-hepatotoxic ingredients were widely scattered in the chemical space. In summary, significant structure–toxicity relationship was observed for hepatotoxicity, indicating the feasibility of assessing the hepatotoxic risk of natural products based on QSAR methods. There was a small amount of overlap between hepatotoxic and non-hepatotoxic ingredients in the chemical space, indicating that some hepatoprotective ingredients collected in the current study may possess potential hepatotoxicity.

**Figure 3:**
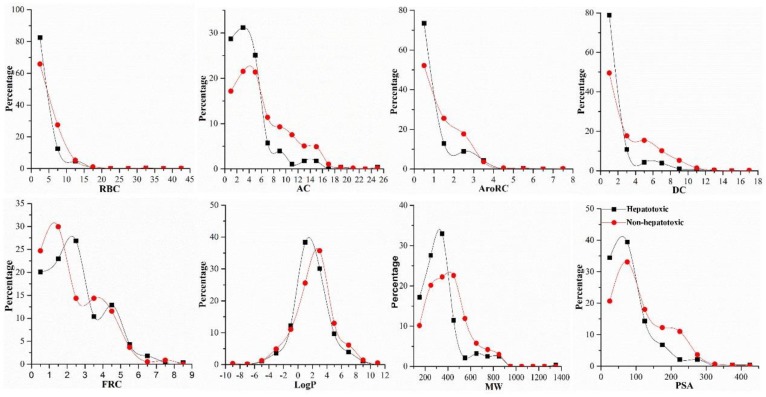
Comparing the chemical properties of hepatotoxic ingredients and non-hepatotoxic ingredients. RBC: the number of rotatable bonds; AC: the number of molecular hydrogen bond acceptor; AroRC: the number of aromatic rings; DC: the number of molecular hydrogen bond donor; FRC: the number of fused rings; LogP: the octanol/water partition coefficient; MW: molecular weight; PSA: the polar surface area.

It has been demonstrated that many physicochemical characteristics are significantly correlated to the ADMET properties of drugs [41,42,43]. The importance of Clog P in assessing the hepatotoxic risk of the synthetic drugs has been confirmed. Chen et al. even proposed that drugs of which dosages ≥ 100 mg/day and logP ≥ 3 are always confronted with higher hepatotoxic risk. They defined this rule as the “rule-of-two” and applied it to estimate the hepatotoxic risk of the synthetic drugs [23]. For natural products, the physicochemical characteristics distribution of hepatotoxic ingredients has not been investigated. Here, comparison between hepatotoxic and non-hepatotoxic ingredients focused on eight physicochemical properties was conducted (Figure 3). In terms of RBC and Log P, there was no significant difference between hepatotoxic and non-hepatotoxic ingredients. Compared to the non-hepatotoxic ingredients, the distribution of AC, DC, MW, PSA, and AroRC of the hepatotoxic ingredients was more concentrated. Similar to the distribution of the hepatotoxic ingredients in the chemical space, significant cluster patterns were also observed in the distribution of these five physicochemical properties. For each of these five properties, the hepatotoxic ingredients were tended to possess smaller property values than the non-hepatotoxic ingredients. In summary, for the distribution of physicochemical property, there was significant difference between hepatotoxic and non-hepatotoxic ingredients. Therefore, we can claim that these properties may be useful in the development of risk measurement model for hepatotoxicity of natural products.

### 2.3. Eight Structural Categories with Higher Frequency of Hepatotoxicity

In Section 2.2.1, we annotated the category and sub-category of each ingredient based on its skeleton. For each sub-category, we investigated its total number of occurrences as well as its frequency in hepatotoxic and non-hepatotoxic ingredients, respectively. Finally, a total of eight sub-categories with higher frequency of hepatotoxicity were identified and detailed in Table 4. Phytochemicals belong to these sub-categories may be confronted with higher hepatotoxic risk.

#### 2.3.1. Pyrrolizidine Alkaloids

Pyrrolizidine alkaloids (PAs) displayed the highest frequency of hepatotoxic ingredients with count of 77 and percentage of 100%. As the largest hepatotoxic group in natural products, severe hepatotoxicity of PAs has long been recognized. It was reported that PAs can induce a series of liver diseases, including hepato-sinusoidal obstruction syndrome and veno-occlusive disease. PAs are produced by plants as a defense mechanism against insect herbivores and are frequently distributed into plants within the families of Fabaceae, Orchidaceae, Asteraceae, and Boraginaceae [44]. Notably, not all PAs are toxic. The esterification with a branched-chain acid and the 1–2 unsaturation are required for the occurrence of hepatotoxicity [45]. Generally, the native form of PAs is non-toxic. It was reported that the active metabolites are contributable to the hepatotoxicity of PAs [46]. PAs always undergo three major biotransformations. Hydrolysis and N-Oxidation do not lead to hepatotoxicity. However, when PAs are biotransformed by CYP3A, various degrees of hepatotoxicity will be observed [47,48]. The mechanisms of PAs-induced hepatotoxicity appear to be related to the binding of the active metabolites and DNA/protein [49,50]. The detailed mechanisms of liver injury induced by PAs have been extensively reviewed elsewhere [51,52,53].

#### 2.3.2. Phenylpropene-Type Simple Phenylpropanooids

Sub-category with ID 2 is phenylpropene-type simple phenylpropanooid with frequency of 9 (eugenol, methyleugenol, acetyleugenol, myristicin, α-Asarone, β-Asarone, safrole, anethole, and estragole) and percentage of 100.00%. Eugenol, methyleugenol, and acetyleugenol, three important natural ingredients from several herbs, are widely consumed in foodstuffs and cosmetics used as flavoring agents and fragrance, respectively [54]. The hepatotoxicity of these three ingredients has been well documented. Mice depleted of hepatic glutathione treated with eugenol (400–600 mg/kg, po) can lead to hepatic congestion, centrilobular necrosis of hepatocytes, and the increase of relative liver weight and serum glutamic pyruvic transaminase. It was generally believed that its hepatotoxicity was attributed to the CYP450-dependent metabolic bioactivation [55]. The formation of quinone methides from eugenol has been extensively studied. It was reported that the covalent modification of DNA mediated by quinone methide can lead to cytotoxic effects, which may contribute to the organ toxicity of eugenol [56]. As the major component of sassafras oil, safrole has a strong antifungal effect [57]. However, it has been categorized into group 2B carcinogens for its severe hepatocarcinogenic [58]. Safrole contains the methylenedioxyphenyl (MDP) structure. Early studies revealed that most MDP agents possess relatively low intrinsic toxicity. However, the reactive metabolites (RMs) from MDP agents can lead to a range of direct or indirect toxicity [59]. In organism, safrole was activated by CYP1A2 and produced several RMs, these RMs were identified as quinone and its tautomers [57]. It has been demonstrated that these RMs may mediate the hepatotoxicity of safrole. Asarones are mainly distributed into some aromatic plants, especially species within the genus of Acorus. In addition to beneficial properties, their potential hepatotoxicity also gained increasing attention in recent years [60]. In one study, significant morphology changes of hepatocytes were observed when the adult rats were exposed to α-asarone (1–2 weeks) [61]. In addition, a cell-based investigation in THLE-2 cells conducted by Patel et al. found that the cytotoxicity of asarones was associated with lipid peroxidation and glutathione depletion, indicating that oxidative stress induced by asarones may be involved in the hepatotoxicity of asarones [62]. Summarily, the hepatotoxicity of compounds within this group was significantly associated with the quinone methide bioactivation pathway.

#### 2.3.3. Benzophenanthridine Alkaloids

Sub-category with ID 3 is benzophenanthridine alkaloid with frequency of 4 (chelidonine, chelerythrine, sanguinarine, and nitidine) and percentage of 80.00%. Chelidonine, chelerythrine, and sanguinarine are more frequently distributed into species in Papaveraceae. The hepatotoxicity of these three ingredients has long been recognized. However, their toxic mechanisms are still far from elucidation. The hepatotoxicity of sanguinarine has been observed in rats which was characterized by the increase of serum aspartate aminotransferase, alanine aminotransferase, lactate dehydrogenase activities, hepatic vacuolization, lipid accumulation, lipid peroxidation and the decrease of triglyceride. Oxidative stress and mitochondrial dysfunction may be two major reasons for its hepatotoxicity. Additionally, the iminiun bond within sanguinarine may play an essential role in the occurrence of its hepatotoxicity [63].

Nitidine is a quaternary ammonium alkaloid isolated from *Zanthoxylum nitidum.* Its hepatotoxicity has been confirmed in primary cultured rat hepatocytes. Nitidine can reduce the cellular viability in a concentration-dependent manner, with an IC50 of 2.83 ± 0.53 uM. Based on Madin-Darby canine kidney cells, Li et al. has explored the contributions of human OCT1, OCT3, and CYP3A4 to nitidine-induced hepatocellular toxicity [64]. It was reported that the hepatotoxicity of nitidine was significantly associated with the uptake of nitidine in hepatocytes mediated by OCT1 and OCT3. However, CYP3A4-mediated metabolism can attenuate the hepatotoxicity induced by nitidine.

#### 2.3.4. Cadinane-Type Sesquiterpenes

Sub-category with ID 4 is cadinane sesquiterpene with frequency of 4 (9-oxo-agerophorone, 2-deoxo-2-(acetyloxy)-9-oxoageraphorone, 9-oxo-10,11-dehydroagerophorone, and gossypol) and percentage of 80.00%. 9-oxo-agerophorone (OA), 2-deoxo-2-(acetyloxy)-9-oxoageraphorone (DAOA), and 9-oxo-10,11-dehydroagerophorone (ODA) were isolated from *Eupatorium adenophorum*. The hepatotoxicity of these three ingredients has been identified very early. Based on Kunming mice, Ouyang et al. evaluated the hepatotoxicity of OA, ODA, and DAOA by acute and sub-acute study. In acute study, compared to OA (1470 mg/kg BW) and ODA (1470 mg/kg BW), DAOA showed the lowest LD50 of 926 mg/kg BW for male mice. In sub-acute test, significant pathological changes of hepatocytes and hepatic lobules were observed under the condition of dose 300 mg/kg BW (for 7 days), and the pathological changes were consistent with hepatic injury and cholestasis [65].

Gossypol is a natural phenol present in the cotton plants (*Gossypium spp.*). In vivo hepatotoxicity of gossypol acetic acid (GAA) was detected against Wistar rats at the dosage of 30 mg/kg daily (2 weeks) and 15 mg/kg daily (4 weeks), respectively. As a result, significant pathological changes of liver cells, including widening of perinuclear space, dilation of endoplasmic reticulum, vacuolation of mitochondria, and proliferation of collagen fibers in Disse’s spaces were observed at both dosages, which indicated that GAA can cause damage to liver cells [66]. The liver toxicity of gossypol was also investigated in lactating Holstein dairy cows at dosage of 1000 mg of gossypol per kilogram of dry matter feed (28 days). In gossypol-treated group, the level of alanine aminotransferase, choline esterase, and glutathione transferase was significantly increased. Inversely, the level of glucose was significantly decreased. Results mentioned above revealed that gossypol induced oxidative stress and hepatotoxicity [67]. Some researchers pointed out that the hepatotoxicity of gossypol can be alleviated by combining with bovine serum albumin (BSA) [68]. However, some other studies reported that gossypol-BSA was not effective to prevent the acute hepatotoxicity of gossypol [69]. Whether gossypol-BSA is effective to avoid the hepatotoxicity of gossypol? To answer this question, a deeper step study is required.

#### 2.3.5. p-Menthane-Type Monoterpenes

Sub-category with ID 5 is p-menthane monoterpene with frequency of 13 (isopulegone epoxide, isopulegol, pulegol, piperitone, piperitenone, (S)-pulegone, (R)-pulegone, isomenthone, Menthone, Menthofuran, d-limonene, menthol, 2-(Propan-2-ylidene) cyclohexanone) and percentage of 76.47%. These monoterpenes are derived from leaves of the plant in Mint genus. During the past decades, many of them were extensively consumed in food, cosmetics, and the medical industry. However, the hepatotoxicity of these ingredients was reported in many cases. Significant coagulative necrosis of centrilobullar hepatocytes was observed in Swiss-Webster mice administered pennyroyal oil at dose of 500 mg/kg. According to the strength of hepatotoxicity, these ingredients can be roughly categorized into low toxicity (Menthone, isomenthone, pulegol, piperitone, piperitenone, and isopulegone), medium toxicity ((S)-pulegone, (R)-pulegone, isopulegone epoxide, and 2-(Propan-2-ylidene)cyclohexanone), and high toxicity (Menthofuran) [70]. The hepatotoxicity of these ingredients was generally reported when they were taken by mouth, indicating that the formation of reactive metabolites may be an important risk factor for the occurrence of their hepatotoxicity. As the major constituent of pennyroyal oil, the content of pulegone is significantly higher than menthofuran. It was reported that menthofuran is the major metabolite of pulegone and is more toxic than pulegone in rats [71]. However, an opposite result was achieved in human [72]. The precision-cut liver slices (PCLS) from five patients were used to test the hepatotoxicity of menthofuran and pulegone. As a result, pulegone changed miRNA expression and decreased PCLS viability more significantly than menthofuran. Inconsistency between these two studies maybe caused by the species difference. Additionally, results obtained by Zárybnický et al. implied that miR-155-5p may be a promising marker of pulegone induced hepatotoxicity [72].

#### 2.3.6. Guaiane-Type Sesquiterpenes

Sub-category with ID 6 is guaiane sesquiterpene with frequency of 9 (Atractyloside A, linifolin A, geigerinin, mexicanin I, helenalin, 6α-hydroxy-2,3-dihydroaromaticin, zedoarondiol, aerugidiol, and dehydrocostus lactone) and percentage of 64.29%. Linifolin A, geigerinin, Helenalin, and mexicanin I are presented in *Helenium aromaticum*. 6α-hydroxy-2,3-dihydroaromaticin is isolated from *Telekia speciosa*. All these five ingredients were potent hydroxyl radical scavengers in vitro. In vivo tests in rats demonstrated that these ingredients can lead to elevation of glutathione peroxidase activity and glutathione level depletion by their pro-oxidative properties [73]. In another study, their effects on several drug-metabolizing enzymes in the liver and the antioxidant enzyme systems were explored [74]. Consequently, the elevation of glutathione peroxidase level was observed in all treatment groups. The activity of glutathione reductase and catalase was elevated only for 6α-hydroxy-2,3-dihydroaromaticin group and mexicanin I group. All these ingredients can decrease the activity of superoxide dismutase with the exception of 6α-hydroxy-2,3-dihydroaromaticin. A total of five drug-metabolizing enzymes in the liver were considered, including CYP450, NADPH-CYP450 reductase, anlinie hydroxylase, anlinie demethylase, and glutathione S-transferase (GST). Linifolin A, helenalin, and mexicanin I reduced the activity of all the five drug metabolizing enzymes. Geigerinin caused decrease of the activity of anlinie hydroxylase, anlinie demethylase, and GST. Inversely, 6α-hydroxy-2,3-dihydroaromaticin can strengthen the activity of anlinie hydroxylase, anlinie demethylase. Merrill et al. have attempted to investigate the role of glutathione in the hepatotoxicity of helenalin and discovered that the hepatotoxicity of helenalin was strongly dependent on the deletion of hepatic glutathione [75]. Zedoarondiol and aerugidiol, two sesquiterpenes, were isolated from *Curcuma zedoaria*. Their cytotoxicity in the hepatocytes was detected by MTT assay [76]. They were also found to can aggravate the acute liver injury induced by D-galactosamine [77].

#### 2.3.7. Emodin-Type Anthraquinones

Sub-category with ID 7 is emodin-type anthraquinone with frequency of 6 (Rhein, chrysophanol, chrysazin, aloe emodin, physcione, and emodin) and percentage of 54.44%. These ingredients were the major hepatotoxic components of *Fallopia multiflora* and several species in genus of Rheum (*Rheum palmatum*, *Rheum offcinale*, and *Rheum tanguticum*). Emodin was the most popular member among these six ingredients. Its hepatotoxicity has been extensively studied, including inflammation, fat vacuole, and hepatocyte apoptosis. Both in Sprague-Dawley (SD) rats and HepaRG cells, hepatotoxicity caused by emodin was observed in a time- and dose-dependent manner. It has been proved that emodin induces liver injury by inhibiting proton transport, inhibiting bile acids transporters, and activating a mitochondrial apoptosis pathway [78,79]. Additionally, GSH depletion and CYP3A induction may be also related to the hepatotoxicity of emodin [80]. Recently, based on quantitative proteomics, L02 cells were adopted to investigate the potential mechanism of emodin-induced hepatocyte apoptosis. The results showed that hepatocyte apoptosis induced by emodin may be dependent on the inhibition of mitochondrial complex function [81]. Hepatotoxicity of emodin in male rats was significantly lower than that in female rats. Such a gender difference may be associated with the coupling of UGT2B7 and MRP2 [18].

It was reported that CYP2C19 could activate rhein to reactive metabolite, which may mediate the hepatotoxicity of rhein. Metabolism of rhein caused production of oxygen-derived free radicals which disrupted the mitochondrial functions and leaded to cell death [82]. In another study, it was reported that rhein can lead to primary human hepatic HL-7702 cells apoptosis via endoplasmic reticulum-stress and elevating intracellular calcium level [83]. Based on HepaRG cells, the cytotoxicity of rhein was also investigated. It demonstrated that rhein induced cell death by activating Fas- and mitochondrial-mediated pathways of apoptosis [84].

Sandwich-cultured rat hepatocytes were employed to investigate the hepatotoxic mechanisms of several anthraquinones. Chrysophanol and physcione were found to can inhibit the bile acids transporters and eventually alert the disposition of bile acids. This was regarded as one of the hepatotoxic mechanisms of these two ingredients [85].

In vitro studies both in HepaRG and HL-7702 Cells indicated that aloe emodin induced apoptosis in a dose- and time-dependent manner. The underlying mechanisms may be involved in mitochondrial pathway and Fas death pathway, which were affected by the generation of reactive oxygen species [86,87]. In another study, a series of pathological changes were observed in the liver tissues of zebrafishes treated with aloe-emodin, including large patches of necrosis, obvious morphological changes, irregular arrangement of liver cells, severe vacuolar degeneration, and local necrosis. The activation of NF-κB inflammatory pathway and P53 apoptosis pathway may be involved in the hepatotoxicity of aloe-emodin in zebrafish [88].

#### 2.3.8. Eudesmane-Type Sesquiterpenes

Sub-category with ID 8 is eudesmane-type sesquiterpene with a frequency of 7 (reynosin, alantolactone, santamarine, α-costol, asperilin, telekin, and santonin) and percentage of 41.18%. Reynosin, alantolactone, santamarine, and α-costol are the major hepatotoxic constituents of *Aucklandia lappa* and *Inula helenium*. Both in vivo and in vitro study indicated that thioacetamide-induced liver injury can be alleviated by reynosin. However, significant hepatotoxicity was also detected when reynosin was used at higher concentrations [89]. The inhibitory effects of alantolactone on CYP450s have been evaluated in human liver microsomes. The activity of CYP3A4 and CYP2C19 was significantly and moderately decreased, respectively. The activity of CYP2A6, CYP2C9, and CYP2D6 was not affected. These results demonstrated that the interaction between alantolactone and drugs metabolized by CYP3A4/CYP2C19 deserves more attention [90]. Asperilin and telekin are from *Telekia speciosa*. In vivo test in rats indicated that they can lead to glutathione level depletion as well as elevation of glutathione peroxidase activity by their pro-oxidative properties [73]. They could also affect the antioxidant enzyme systems. Both asperilin and telekin increased the hepatic activity of glutathione peroxidase in vivo in rats. The activity of glutathione reductase and catalasewas was only increased by asperilin. The increased of superoxide dismutase was solely observed for telekin [74].

### 2.4. Structural Alerts for Hepatotoxicity

It has been demonstrated that some substructures were related to the organ toxicity of drugs. Through a comprehensive analysis for the mechanism-based inhibitors from phytomedicine, Wang et al. identified two potential hepatotoxic substructures, amine and furan heterocycle [91]. However, it is always time consuming and labor intensive to identify structural alerts (SAs) manually. In addition, some important SAs may escape from our sight owing to the limited computing power of the human brain. With the development of computational chemistry, automatic extraction of SAs based on the QSAR methods has gained increasing attention. Compared to identify SAs manually, automatic extraction of SAs is more efficient [92,93]. 

Structural fragments responsible for the activity/toxicity under investigation are always called as SAs, which cannot only help to assess the activity/toxicity of xenobiotics but also assist to decipher the biochemical mechanism of action [94]. Here, SARpy software was utilized to extract SAs for hepatotoxicity automatically [95]. SARpy is very effective in detecting SAs and has been successfully applied to many prior studies [96]. Fragmentation of the chemical structure is the first step of SARpy by which it attempts to generate a series of structural fragments. Then the prediction performance of each structural fragment against the dataset under investigation was evaluated, and the structural fragments with higher predictive power were selected as SAs. SARpy measures the predictive power of each structural fragment by the likelihood ratio (LR). A higher LR value always represents a higher predictive power.

Compounds collected in Section 2.2.1 were used as input to run SARpy within different settings (max, optimal, and min), and the minimum number of occurrences for the SAs was set to 5. As a result, a total of 15 SAs for hepatotoxicity were identified and detailed in Table 5 where the representative compound and distribution of each SA were also available. 

According to the distribution of SAs, these SAs can be divided into five groups, alkaloids (1, 3, 4, 5, 9), terpenoids (2, 6, 7, 10, 12, 13, 14), mixture of terpenoids and alkaloids (8), emodin anthraquinones derivatives (15), and furanocoumarins (11). Several of these SAs were only existed in specific compound families. SAs with ID of 1, 3, and 4 were only found in pyrrolizidine alkaloids. SAs with ID of 15 and 11 were the core structures of emodin anthraquinones derivatives and furanocoumarins, respectively. 

These SAs may be related to the occurrence of hepatotoxicity. Actually, it has been proved that compounds containing SAs of ID 1 or ID 13 were more frequently suffered from hepatotoxicity. Requirements for the hepatotoxicity of PAs seemed to be including 1-2 unsaturation bond and an esterified hydroxyl group [45]. PAs containing 1-2 unsaturation bond undergo biotransformation mediated by CYP450 enzymes and eventually are metabolized to “pyrrole type esters”, which responsible for PAs-induced hepatotoxicity. Saturation of the double bond always alleviates or even eliminates the toxicity [97]. Esterification is not a decisive factor for PAs-induced hepatotoxicity. Its role is to enhance the solubility of PAs in the body [47]. The hepatotoxicity of several monoterpenes isolated from pennyroyal oil was investigated by Gordon et al. [98]. An interesting finding was that α-isopropylidene ketone unit significantly affected the hepatotoxicity of pulegone analogues. Reduction of either the ketone group or the isopropylidene double bond decreased the hepatotoxicity of compound dramatically. In addition, SAs of ID 1 and ID 11 were consistent with the two potential hepatotoxic substructures identified by Wang et al. (amine and furan heterocycle) [91]. Cases mentioned above indicated the reliability of SAs identified in this work. In summary, a total of 15 SAs were identified which will provide valuable information for the assessment of hepatotoxic risk and the elucidation of hepatotoxic mechanism.

### 2.5. Construction of Herb-Ingredient Network

**Figure 4:**
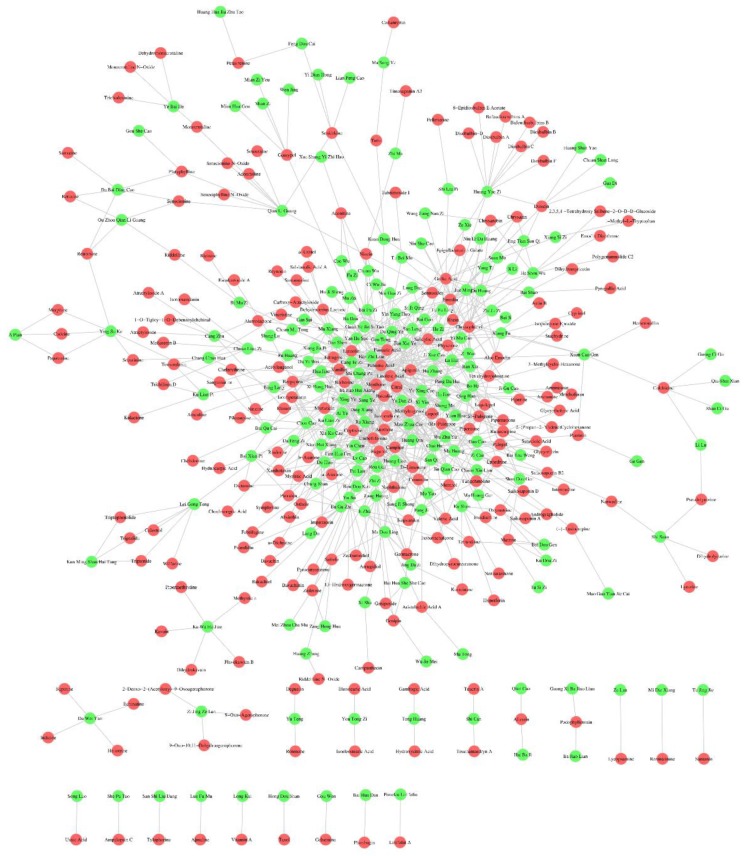
Herb-ingredient network. The green and red circle nodes represented the hepatotoxic herbs and hepatotoxic ingredients, respectively. The gray edges between the herbs and ingredients indicated that the herbs include the hepatotoxic ingredients.

As is well known to all, herbs produce their efficacy/toxicity through the synergistic effects of multi-ingredients, multi-targets, and multi-pathways. Research focused on single ingredient is difficult to explain the activity/toxicity mechanisms of herbs comprehensively. A systemic investigation into the active/toxic ingredients of herbs may help to elucidate their active/toxic mechanisms. In this work, a series of hepatotoxic medicinal plants (335) and hepatotoxic ingredients (296) were collected. Did any relationships exist between these medicinal plants and ingredients? To answer this question, a relation network between these medicinal plants and ingredients was constructed against the “herb-ingredient” pair data included in three typical herb databases (TCMSP, TCMID, and ETCM). As demonstrated in Figure 4, the herb-ingredient network included 410 nodes and 808 edges. A total of 223 hepatotoxic ingredients and 187 hepatotoxic herbs were involved. This herb-ingredient network can be used to identify the hepatotoxic ingredients of hepatotoxic herbs quickly and comprehensively. A detailed description of the herb-ingredient network was provided in Supplementary file 4. 

**Figure 5:**
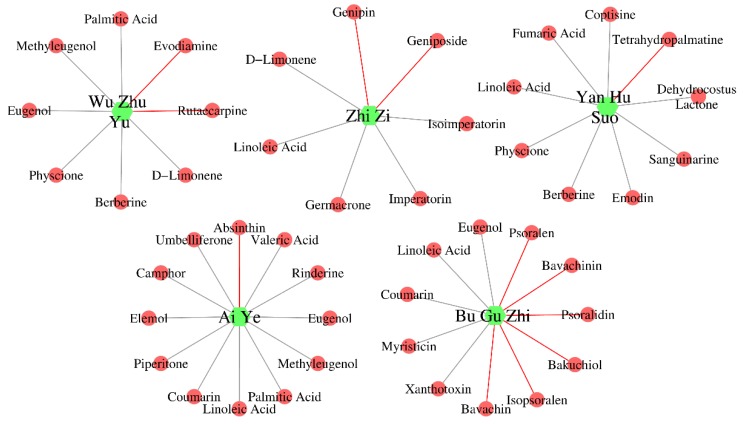
Hepatotoxic ingredients identified by the herb-ingredient network for Wu Zhu Yu, Zhi Zi, Yan Hu Suo, Ai Ye, and Bu Gu Zhi. The green hexagons and red circles indicated hepatotoxic herbs and hepatotoxic ingredients, respectively.

Taken five commonly used hepatotoxic herbs (Zhi Zi, Yan Hu Suo, Bu Gu Zhi, Ai Ye, and Wu Zhu Yu) as cases, we validated the reliability and validity of this herb-ingredient network. Figure 5 displayed the hepatotoxic ingredients of each hepatotoxic herb mentioned above. The green hexagons and red circles indicated hepatotoxic herbs and hepatotoxic ingredients, respectively. The red edges indicated that the hepatotoxic ingredients of the corresponding herbs can be identified directly via literature retrieval. For those herb-hepatotoxic ingredients relationships, which cannot be identified directly by literature retrieval, we displayed the corresponding edges with gray. For Zhi Zi, a total of seven hepatotoxic ingredients were identified. Genipin and geniposide can be identified directly by literature retrieval [99,100]. The other five hepatotoxic ingredients (Isoimperatorin [91], linoleic acid [101], imperatorin [91], germacrone [102], and d-limonene [102]) were identified by our herb-ingredient network. For the other four hepatotoxic herbs, the number of hepatotoxic ingredients was 9 (Yan Hu Suo), 11 (Bu Gu Zhi), 12 (Ai Ye), and 8 (Wu Zhu Yu), respectively. The count of hepatotoxic ingredients identified by our herb-ingredient network for the corresponding hepatotoxic herbs was 8, 5, 11, and 6, respectively. All of the hepatotoxic ingredients reported in the literature for each of these five hepatotoxic herbs were included in our results, indicating the reliability and validity of our herb-ingredient network.

## 3. Materials and Methods

### 3.1. Search and Identification Terms

To identify publications to be included in the current study, we retrieved the PubMed and CNKI (China National Knowledge Infrastructure) databases using key phrases listed as follows: Traditional Chinese Medicine, TCM, herbal, herb, medicinal plant, and botanical. These terms were used as search terms combined with hepatotoxicity, liver toxicity, liver failure, liver injury, liver damage, hepatitis, liver cancer, liver tumor, hepatocellular carcinoma, liver cirrhosis, hepatomegaly, liver neoplasms, fatty liver, hepatoma, liver fibrosis, jaundice, cholestasis, liver protection, hepatoprotective, and hepatoprotection. The retrieval time span was limited to 2009–2018. By reviewing the scientific literature one by one, we collected hepatotoxic herbs, hepatotoxic ingredients, and hepatoprotective ingredients, respectively. To ensure the reliability of the data, each record was checked by two individuals simultaneously. Additionally, two comprehensive databases related to DILI, LiverTox [4] and Hepatox, were also retrieved to collect herbs/phytochemicals with potential hepatotoxicity. Of note, the hepatoprotective ingredients without any hepatotoxic reports were used as non-hepatotoxic ingredients in the following analysis.

Latin name and taxonomy information about each hepatotoxic plant were retrieved from the NCBI (National Center for Biotechnology Information) taxonomy database [103]. The simplified molecular-input line-entry system (SMILES) information and CAS number of each compound were collected from the PubChem database by name matching.

### 3.2. Phylogenetic Tree Construction

The TAX ID of each hepatotoxic plant was identified by retrieving NCBI taxonomy database. Then phyloT, a NCBI taxonomy-based phylogenetic tree generator, was utilized to construct phylogenetic tree with the TAX ID of each hepatotoxic plant used as input. Finally, all of the phylogenetic trees generated in the current study were visualized by iTOL version 4 [104].

## 4. Conclusions

As the natural source of drugs and dietary supplements, herbs play an essential role in drug discovery and development. During the past decades, hundreds of novel drugs and health care products derived from herbs directly or indirectly were approved by China Food and Drug Administration (CFDA) or Food and Drug Administration (FDA) [105,106,107]. An investigation into the distribution of drug-productive species against the phylogenetic tree indicated that most novel drugs are derived from preexisting drug-productive families [108]. In another research, the authors reported that species with close taxonomic relationship always tend to possess similar medicinal properties [109,110,111]. These instances laid foundation for assessing the hepatotoxic risk of medicinal plants via phylogenetic tree analysis. Theoretically, medicinal plants with potential hepatotoxicity should be concentrated in some families rather than distantly distributed in phylogenetic tree. In this work, the distribution of 335 hepatotoxic medicinal plants was investigated against the phylogenetic tree of the mesangiospermae clade. As a result, a significant cluster pattern was observed in order, family, and genus scale, respectively. Medicinal plants with potential hepatotoxicity tended to have closer taxonomic relationship rather than distributed in phylogenetic tree irregularly. Therefore, we can claim that there was certain association relationship between the hepatotoxicity of medicinal plants and their taxonomic positions. Medicinal plants close to hepatotoxic medicinal plants may be faced with higher hepatotoxic risk. Summarily, the hepatotoxic medicinal plants list provided in this work will help to assess the hepatotoxic risk of medicinal plants through phylogenetic tree analysis. 

In some Asian countries, many people always believed that herbal medicine is safe and harmless. However, several commonly used herbs have been reported to cause a series of adverse effects in recent years [112,113,114,115,116]. Among the toxic effects of herbs, hepatotoxicity is one of the leading concerns. Hepatotoxicity always leaded to a series of clinical adverse events, including hepatitis, hepatic fibrosis, liver failure, and even death [1,2,3]. Currently, DILI has been deemed as one of the major factors of the failure of drug discovery [5]. Therefore, risk assessment focused on hepatotoxicity in the early stage of drug discovery is indispensable. However, it is always time-consuming and highly cost to detect the hepatotoxicity of drug candidates by experiment. With the steady accumulation of data related to DILI and the rapid development of computational chemistry, predicting the hepatotoxic risk of drug candidates by in silico methods has gained increasing attention in recent years. Compared to the experimental methods, in silico methods are much faster and cheaper. Currently, there have been many in silico models focused on predicting the hepatotoxic risk of drug candidates in the literature [117,118]. However, almost all of these models were developed solely based on the synthetic drugs. As we all know, the chemical space of natural products is quite different from that of the synthetic drugs. The distribution of the synthetic drugs in the chemical space is relatively compact, while the distribution of natural products is more dispersed [119,120]. In addition, the elementary composition of natural products is also different from that of the synthetic drugs. The synthetic drugs are rich in N atoms. However, natural products tend to contain more O atoms [121]. Therefore, in silico models developed solely based on the synthetic drugs may have limited predictive capabilities for HILI. By incorporating the use of three machine learning algorithms and twelve types of molecular fingerprints, Ai et al. developed an ensemble in silico model for predicting DILI based on 1241 diverse compounds. For ease of access, the authors also provided a user-friendly web server [122]. To the best of our knowledge, this is the latest publicly accessible prediction model for DILI. Based on this in silico model, we evaluated the hepatotoxic risk of each compound collected in this work (296 hepatotoxic ingredients and 584 non-hepatotoxic ingredients). As a result, the overall concordance between the predicted results and the results reported in the literature was only 45.23%. It indicated that in silico models developed solely based on the synthetic drugs is not applicable for natural products. In a recent study, it was reported that adding natural products data into the modeling dataset can improve the performance of in silico model when predicting the hepatotoxicity of natural products [123]. Currently, an in silico model for predicting HILI was still unavailable. The lack of a large scale dataset related to HILI is one of the leading causes. Herein, we provided a large scale dataset of HILI which will lay foundation for the construction of in silico models for predicting HILI. 

The distribution of hepatotoxic and non-hepatotoxic ingredients in the chemical space was investigated. Most hepatotoxic ingredients gathered together. Significant difference of the physicochemical properties between hepatotoxic and non-hepatotoxic ingredients was also detected. These results indicated that it is feasible to assess the hepatotoxic risk of natural products based on the QSAR methods.

For each compound collected in this work, we annotated its structural category and sub-category. The structures of hepatotoxic ingredients were diverse and complex, which involved in a series of sub-categories. Alkaloids and terpenoids were found to be the two major structural categories with frequency of 127 (42.91%) and 88 (29.73%), respectively. Taken several major sub-categories as cases, we summarized and analyzed their hepatotoxicity from the perspectives of structure, pathological characteristic, and mechanism, respectively. There were some chemical bonds/groups significantly associated with the hepatotoxicity of phytochemicals, including the 1–2 unsaturation in PAs, the MDP structure of safrole, the iminiun bond within sanguinarine, and the α-isopropylidene ketone unit within pulegone analogues. The pathological characteristics of HILI were complex and diverse, including morphology changes and apoptosis of hepatocytes, hepatic congestion, inflammation, fat vacuole, and centrilobular necrosis of hepatocytes. Actually, HILI cannot be viewed as a single disease entity, but a spectrum of liver diseases. Generally, it can be roughly categorized into two classes, “intrinsic” and “idiosyncratic”. Intrinsic HILI is predictable, reproducible, and dose-dependent. Idiosyncratic HILI is not clearly dose related but host dependent. Therefore, it is often difficult to detect it by the regulatory animal toxicity studies. Many different mechanisms were reported to be associated with intrinsic HILI. Some drugs were activated by the drug-metabolizing enzymes and metabolized to minor electrophilic metabolites. These highly reactive metabolites depleted glutathione and initiated covalent binding to cellular proteins/DNAs, resulting in lipid peroxidation, disrupting the mitochondrial functions, activating oxidative stress and endoplasmic reticulum stress, and eventually triggering cell death pathways. Drugs decreasing the activity of drug-metabolizing enzymes always resulted in the accumulation of toxic metabolites and leaded to liver injury. The disruption of bile acid homeostasis is another mechanism of HILI. By regulating the expression or localization of bile acids transporters, some herbs or their reactive metabolites inhibited the clearance of bile acids and resulted in hepatocyte damage and cholestasis. In addition, the activation of Fas-, P53-, and mitochondrial-apoptosis pathways also play an essential role in the development of HILI. Idiosyncratic HILI is a rare disease which mainly depends on the host genetic, immunologic, and metabolic factors. Many drug-metabolizing enzymes are highly polymorphic and there are interindividual variability in their metabolic activity. Relatively low metabolic activity always resulted in the accumulation of drugs or their metabolites in the body and leaded to liver toxicity. In addition, some drugs or their reactive metabolites generated autoantibodies by binding to the endogenous proteins. The formation of autoantibody led to hepatocyte death. In summary, the mechanism of HILI is rather complicated. Currently, the hepatotoxic mechanisms for most herbs are far from elucidation. Scientific studies focused on uncovering the molecular basis and mechanism of HILI deserve more attention.

Based on SARpy software, we identified 15 SAs for hepatotoxicity. Two of these structural alerts, ID 1 and ID 12, have been proved to be significantly associated with the occurrence of hepatotoxicity. These SAs cannot only assist to decipher the hepatotoxic mechanisms of herbs but also can help to construct in silico models for predicting HILI. 

Finally, a herb-ingredient network was constructed for the first time against the “herb-ingredient” pair data included in TCMSP, TCMID, and ETCM databases. This herb-ingredient network described the relationship between hepatotoxic herbs and hepatotoxic ingredients. It can be used to identify the hepatotoxic molecular basis of herbal/herbal formula quickly and comprehensively. 

Although some herbs or their ingredients were reported to possess potential hepatotoxicity, one cannot deny their significant efficacy in treating many complex diseases [124,125,126]. In many cases, their toxicity can be alleviated or eliminated through modifying structure, changing the dosage form, or combining with other herbs/ingredients [127,128,129]. Nevertheless, herbs or their ingredients with hepatotoxicity should be carefully administered in clinical application. Generally, damage to the liver will be brought under control after stopping the herbal medication. Avoiding high doses and long term use of herbals may be an effective strategy to reduce the incidence of HILI [53,130,131]. In the future, a comprehensive and systematic database for HILI may help physicians and pharmacologists to reduce HILI.

Nevertheless, we must acknowledge limitations of this work as follows: (1) although a comprehensive literature retrieval was conducted. There may still exist some hepatotoxic ingredients and hepatotoxic medicinal plants escaped from our vision. (2) The completeness of the results provided by our herb-ingredient network is highly depended on the “herb-ingredient” pair data included in the databases. In this work, the herb-ingredient network was constructed against three typical herb databases. With the introduction of more comprehensive “herb-ingredient” pair data, the results provided by our network will be more completeness. (3) The majority of the data used in the current study were not derived from clinical case reports or observational studies but from animal/cell experiments. In the future, with the accumulation of clinical data of HILI, we believe studies focused on HILI will make great progress.

## Figures and Tables

**Table 1:** The 12 orders containing hepatotoxic herbs greater than 10.

Rank	Order	Size	Frequency of Hepatotoxic Herb	Percentage
1	Ranunculales	2830	33	1.1661%
2	Boraginales	2700	12	0.4444%
3	Piperales	4170	11	0.2638%
4	Sapindales	5700	15	0.2632%
5	Apiales	5489	12	0.2186%
6	Gentianales	17000	23	0.1353%
7	Malpighiales	16000	20	0.1250%
8	Caryophyllales	12000	15	0.1250%
9	Asterales	26870	31	0.1154%
10	Fabales	20000	21	0.1050%
11	Lamiales	23800	23	0.0966%
12	Asparagales	36200	14	0.0387%

Data of the order size was derived from Encyclopaedia Britannica.

**Table 2:** Top-10 most productive families of hepatotoxic herbs.

Rank	Family	Size	Frequency of Hepatotoxic Herb	Percentage
1	Aristolochiaceae	590	8	1.3559%
2	Polygonaceae	1100	11	1.0000%
3	Ranunculaceae	2252	16	0.7105%
4	Boraginaceae	2700	9	0.3333%
5	Lamiaceae	7000	17	0.2429%
6	Apiaceae	3700	8	0.2162%
7	Euphorbiaceae	6745	11	0.1631%
8	Asteraceae	26870	31	0.1154%
9	Fabaceae	20000	21	0.1050%
10	Rubiaceae	13150	11	0.0837%

Data of the family size was from Encyclopaedia Britannica.

**Table 3:** The 15 genera containing hepatotoxic herbs greater than 3.

ID	Genus	Frequency of Hepatotoxicity Herb
1	Aristolochia	6
2	Senecio	5
3	Uncaria	5
4	Rumex	5
5	Aconitum	4
6	Actaea	4
7	Epimedium	4
8	Senna	4
9	Gentiana	4
10	Euphorbia	4
11	Symphytum	4
12	Gossypium	4
13	Curcuma	4
14	Dioscorea	4
15	Eupatorium	4

**Table 4:** Eight sub-categories with higher frequency of hepatotoxicity.

ID	Description	Representative Compounds		Hepatotoxic/Total (Percentage)
		Name	Structure	
1	Pyrrolizidine alkaloids	Senecionine	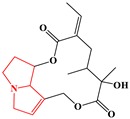	77/77(100.00%)
2	Phenylpropene-type simple phenylpropanooids	Eugenol	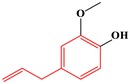	9/9(100.00%)
3	Benzophenanthridine Alkaloids	Nitidine	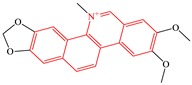	4/5(80.00%)
4	Cadinane-type sesquiterpenes	9-Oxo-agerophorone	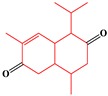	4/5(80.00%)
5	P-menthane-type monoterpenes	Pulegone	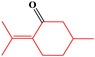	13/17(76.47%)
6	Guaiane-type sesquiterpenes	Atractyloside A	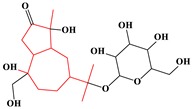	9/14(64.29%)
7	Emodin-type anthraquinones	Rhein	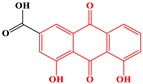	6/11(54.55%)
8	Eudesmane-type sesquiterpenes	Alpha-costol	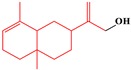	7/17(41.18%)

Total and hepatotoxic indicated the occurrences of the sub-category in the whole dataset and in the hepatotoxic dataset, respectively.

**Table 5:** Fifteen structural alters for hepatotoxicity.

ID	SAs	LR	Hepatotoxic/Total (Percentage)	Distribution of SAs
1	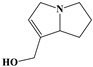	inf	47/47 (100.00%)	Pyrrolizidine alkaloids
2	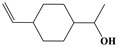	inf	7/7 (100.00%)	Sesquiterpenes
3	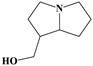	inf	7/7 (100.00%)	Pyrrolizidine alkaloids
4	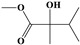	112.77	55/56 (98.21%)	Pyrrolizidine alkaloids
5	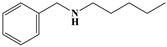	12.30	6/7 (85.71%)	Alkaloids
6	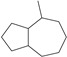	12.30	6/7 (85.71%)	Terpenoids dominated by Guaiane sesquiterpenes
7	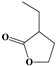	11.62	17/20 (85.00%)	Terpenoids
8	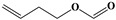	8.71	68/84 (80.95%)	Pyrrolizidine alkaloids and Sesquiterpenes
9	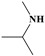	6.66	78/102 (76.47%)	Alkaloids
10	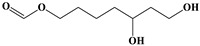	5.33	13/18 (72.22%)	Terpenoids
11	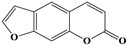	5.13	5/7 (71.43%)	Furanocoumarins
12	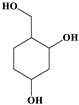	5.13	10/14 (71.43%)	Terpenoids
13	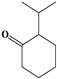	4.10	10/15 (66.67%)	Terpenoids dominated by tetracyclic triterpenoids
14	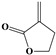	4.10	12/18 (66.67%)	Sesquiterpenes
15	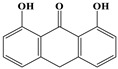	3.69	9/14 (64.29%)	Emodin anthraquinones and their derivatives

Total and hepatotoxic indicated the occurrences of the SAs in the whole dataset and the hepatotoxic dataset, respectively. LR was adopted to evaluate the precision of each SA in differentiating hepatotoxic and non-hepatotoxic ingredients.

## Supplementary Materials

The following are available online at https://www.mdpi.com/1422-0067/20/15/3633/s1.
